# The Clinical Examination of Stools
1A paper read before the Bath Clinical Society.


**Published:** 1915-06

**Authors:** G. Hely-Hutchinson Almond

**Affiliations:** Honorary Pathologist to the Royal Mineral Water Hospital, Bath; Hon. Assistant Pathologist to the Royal United Hospital, Bath


### THE CLINICAL EXAMINATION OF STOOLS.[1]

BY

G. HELY-HUTCHINSON ALMOND, M.A., B.M., B.Ch. (Oxon.), M.R.C.S. (Eng.), L.R.C.P. (Lond.),

*Honorary Pathologist to the Royal Mineral Water Hospital, Bath ; Hon. Assistant Pathologist to the Royal United Hospital, Bath.*

---

REGARDED from a clinical standpoint, the examination of stools is a method of investigation that does not always receive the attention it deserves. A positive diagnosis of disease of the alimentary canal or its appendages, functional, catarrhal, or malignant, may be withheld solely from neglect of fæcal analysis. Moreover, although our knowledge of the ultimate effects of intestinal bacterial activity is not large, some striking illustrations of the possibilities of treatment as the result of this line of research have already been demonstrated.

I propose to describe a routine method of examination, drawing attention only to the more important tests at our disposal, and indicating briefly the significance of each step as it is taken. A specimen should be selected with some care, as free from contamination as possible, and it should for preference be collected in a clean, wide-mouthed, tightly-stoppered glass bottle with a capacity of from four to six ounces. The colour, reaction, smell and physical condition should be noted, and the presence or absence of any extraneous matter. The normal *colour* is due to stercobilin—a derivative of biliary pigment—and to the nature of the food. It is modified by certain drugs, by blood, by the state of the alimentary secretions and mucosa, the nature of the flora, the reaction, and the quantity of

[1] A paper read before the Bath Clinical Society.

moisture. Meat, red wine, and dark-red fruit berries make the colour darker, vegetable fibre paler, chlorophyl greener or more olive, milk diet white or pale orange. Chocolate, logwood, rhubarb, senna, santonin and gamboge have a tendency to turn it reddish brown, ferric iron very dark, ferrous dark only after exposure to the air, bismuth usually a slaty, sheeny grey-black. Blood if reduced and plentiful darkens the colour, and if excessive it gives it the appearance of tar. The bile pigments bilirubin and biliverdin if not reduced turn the motion green; this occurs in lienteric diarrhœa, as the result of the action of certain bacteria, from the action of calomel, and from the presence of bismuth in certain cases. A loose, watery stool is, *ceteris paribus*, paler than a hard, constipated one; an acid one may be pale from the presence of gas bubbles, demonstrable by the fact that it will float; alkalinity has a tendency to turn it reddish brown; fat, if largely unabsorbed, makes the stool pale and pasty; if bile is absent emulsification is retarded, hence the grey-white stool of jaundice; if the pancreatic juice fails, saponification is interfered with, and the stool is pale and yellow; if food is not absorbed from mucous catarrh, as in sprue, the colour is pale or orange-red.

Normally the *reaction* to litmus is amphoteric. Fermentation produces acidity, putrefaction alkalinity. The characteristic *odour* is due to indol, skatol, and perhaps also methyl mercaptan (Herter). It is more pronounced with a meat diet, still more so with putrefaction, in infants it is very faint or absent. A fermented stool has an acid smell due to the presence of volatile fatty acids (butyric, formic, etc). Mucus in large quantities gives it the odour of serum, dysentery that of lime. A cholera stool is odourless.

The further investigation with the naked eye should be directed to discovering the microscopic remains of food stuffs, of mucus, pus, concretions and intestinal parasites.

If the stools are fluid they should be carefully looked at before they are emptied out of the glass bottle, because frequently small opalescent masses of mucus can be seen lying at the bottom on the surface of the glass. The most convenient method of search for heterogeneous particles is by transferring from one to three grams of fæces on to a glass plate, the under surface of which has been painted one-third black and one-third white. A little saline or water is then added, and the fæces are then rubbed down with a pestle or spatula. Fragments of meat-fibre, starch, cellulose, fat, connective tissue, mucus, pus, "sand" or other suspicious particles should if present be picked out for microscopical examination. "Sand," if found, may be collected by shaking up the final remainder of the stool in the glass bottle with repeated washings of water, and gathering it from the surface or from the bottom of the vessel, according as it sinks or floats.

A microscopical examination is in many respects of greater importance than that of the naked eye. For since masses of food which have been imperfectly masticated and bolted may escape the action of the alimentary enzymes, the presence of excessive amounts of unaltered food in the homogeneous parts of the stool may offer direct evidence of their absence. In proceeding, a platinum loopful of a homogeneous portion should be put on to a glass slide and a cover glass well pressed down so as to make a thin, transparent smear. Examination will reveal a considerable quantity of pale, amorphous detritus. Search should be made for food remains, mucus, inorganic granules, crystals, etc.

MUSCLE FIBRES can be easily recognised by their fine cross striations. They may be present as small, detached square or oblong fragments, or in masses clustered together or ranged in parallel. Their presence in any but minute quantities denotes one of the three following conditions :—

1. Rapidity of the passage of food through the intestinal tract, such as occurs in diarrhœa or after the administration of a purgative.

2. Deficiency of hydrochloric acid in the gastric juice. The connective tissue surrounding the fibres remains unacted upon, and hence the proteolytic enzymes are unable to penetrate to the muscle fibres. In such cases careful naked-eye examination should be made on the plate for connective tissue, which, over the dark ground, is seen to have a fibrous structure, which clears up on the addition of acetic acid. It may be distinguished from mucus by its firmer structure.

3. Deficiency or absence in the alimentary canal of the pancreatic juice. In such cases the nuclei may be well preserved, and should be stained with millon followed by the application of heat or with methylene blue.

VEGETABLE REMAINS.—*Cellulose* in the form of spirals or cellular shells are quite common, so also are small vegetable hairs formed of a homogeneous material with a well-marked central canal. If *starch* is suspected Lugol's solution should be run under the slide, or another preparation mixed with the solution should be prepared. Clear differentiation must be made from starch granules and small particles of black due to charcoal, iron or bismuth which may be present, and which should have been previously observed. The presence of starch in any quantity is one of the indications of pancreatitis, and it is also present in the stools of certain children who do not thrive as a result of pancreatic deficiency, a fact that can be rectified by treatment with diastase or malt.

FAT in some form or other is invariably present in the stools. Occasionally there may be large fat concretions, and both in diarrhœa and in pancreatitis small masses that can be detected by the naked eye are by no means rare. In certain conditions to be considered later the stools may be pasty and obviously fatty.

Microscopically in the normal stool small glistening beads of neutral fat may be usually made out. In order to identify them half a gram of fæces should be put into a test tube, a drop of acetic acid added if the stools are alkaline or neutral, and one c.c. of a saturated solution of *Sudan* iii. in 70 per cent. alcohol poured in and the mixture well shaken up and allowed to stand for some hours. When examined, the fat globules will be recognised by their bright pink appearance, while fatty acids will be yellow, and soaps a pinkish yellow. Fatty acids can, however, be easily seen in a fresh smear by the peculiar shape of their needles, and masses of these crystals can be seen clustered together in cases of pancreatitis and in sprue.

The normal quantity of fat in a healthy stool is from 25 to 33 per cent. of the dried solid constituents. The proportion of neutral fat and free fatty acid on the one hand and combined fatty acids or soaps on the other is roughly half and half. In certain diseases of the liver, pancreas and alimentary canal both the total quantity may be considerably increased and the proportions altered, and if this should be the case it is of the utmost importance that a quantitative and proportional estimate should be made.

For this purpose from 20 to 30 grams of the fæces are dried in a porcelain dish on a water bath in a fume cupboard until absolutely dry. The dish is then transferred to a desiccator, and the dried fæces are then ground to a fine powder in a mortar.

Two 50 c.c. measured glass tubes are then taken, and an accurately-weighed half gram of the powder is placed in each tube. Into one of them 10 c.c. of 33 per cent. HCl is then poured, and the mixture heated on a water bath for half an hour. All the combined fatty acids, or in other words the soaps, are by this method converted into free fatty acids. The mixture is then cooled and methyl ether is run in up to the 50 c.c. mark.

Into the other tube 10 c.c. of distilled water is run, and then ether to the 50 c.c. mark. The ether in this case only takes up the fat and free fatty acids, whereas in the first tube all the fatty acid radical present is dissolved. Twenty c.c. of the ether extract is pipetted off into a weighed glass beaker, and the ether evaporated off and the beaker weighed again.[1] This is done in each case. In working out the quantities an allowance of 2 c.c. of ether dissolved in the 10 c.c. of HCl or water must be made, *i.e.* the total ether added must be reckoned as 38 per cent. and not 40.

CRYSTALS of the alkaline earths are commonly seen under the microscope. Calcium and ammonio-magnesium or triple phosphates are the more usual. Calcium oxalate is found especially after large vegetable diet, such as spinach, etc.

INTESTINAL "SAND" is fairly frequent in mucous colitis, and varies in colour from a brick red to a dirty white. The particles are composed chiefly of calcium phosphate, with traces of calcium oxalate, salts of magnesium, iron silicates, and organic matter, etc., coloured with stercobilin. "Sand" also exists in the form of calcium soap, and both these forms have to be differentiated from "vegetable sand," which usually floats, and which has no pathological significance. It may consist of the seeds of fruits or other small masses of wooden fibre.

INTESTINAL CONCRETIONS are not often found, but should be searched for. Biliary concretions disintegrate easily. They are usually laminated, and are formed mainly of cholesterin and calcium-bilirubin. Pancreatic concretions (calcium carbonate) are rare. Concretions may also be formed from substances taken by the mouth, *e.g.* magnesium

[1] The beaker in each case having been carefully dried in the dessicator.

carbonate and phosphates, bismuth, salol, shellac and occasionally fat, especially in oil cures.

Intestinal Parasites should in all suspected cases be searched for. If not actually found the presence of their eggs would denote their existence. Eighty-one species are known to infect man.

Endomyces have been obtained from the stools of normal individuals and those suffering from chronic intestinal diseases (sprue, etc.). Castellani has isolated no less than six varieties from stools and intestinal scrapings.

Mucus may be secreted from any part of the small or large intestine. A coating of mucus to a well-formed stool denotes an origin in the rectum or pelvic colon, or, if mixed intimately, higher up in the large or small intestine. In the case of loose stools this generalisation fails, but it may be usually inferred that the smaller the particles of mucus the higher is their origin. Macroscopically mucus may resemble small particles of fat or hyaline vegetable matter. Macroscopically it has the appearance of long streaky granular semi-transparent beads. For confirmation a bead of mucus should be spread out on a slide, fixed and stained with methylene blue. If leucocytes are present in any numbers a catarrhal condition at its seat of formation may be surmised.

Pus, if present in considerable quantities, points to some perforating abscess. If the cells are only to be found in small amount and by the aid of the microscope, the probability is that there is some ulceration ; but the absence of pus-cells does not preclude an ulcer, because the cells are apt to decompose and fragment.

Occult Blood.—If malignancy is suspected the stools should be searched for small masses of growth, which if found should be fixed and stained. Such findings are, however, rare, and by far the most important procedure is the test for occult blood.

The guaiacum test should first be applied, and if this is positive the benzidene (Schlesinger and Holst's method).

If the latter is negative no occult blood is present, though it is just as well to do a control.

A diet of under 40 grams of meat is not sufficient to give the benzidene reaction, but a special meat free diet for three previous days is to be preferred. The benzidene perborate method (Colwell and MacCormac) will give a positive reaction with the minutest traces of blood (one part in a million), and which will not react to any enzyme or to iodide of potash, and which will, moreover, react even if inhibitory substances are present. In any doubtful case this test should certainly be performed.

In suspected gastric or duodenal ulceration the test should be undertaken (if negative) on several different occasions.

*Guaiacum Test.*—Extract with glacial acetic acid. Add equal parts of ether. Take some of the ether solution floating on the top and mix with equal parts of water. Add a few drops of tincture guaiacum and then ozonic ether. Positive test, possibly blood, but may be due to pus, saliva or organic matter.

*Benzidene Test.*—Take a piece of fæces the size of a walnut. Add two or three times the quantity of water. Boil and cool. Filter if necessary, and add to benzidene solution. Benzidene solution. Add a knife point of benzidene (Merck) to 2 or 3 c.c. of glacial acetic acid. Add 5 to 10 drops to 3 c.c. of 3 per cent. solution of $H_2O_2$ so as to make mixture opalescent.

*Benzidene Perborate Test.*—Mix 0.1 gram of benzidene, 0.1 of sodium perborate in 10 c.c. of glacial acetic acid. Take 5 c.c. of liquid fæces (or half gram of solid) and add

5 c.c. of glacial acetic acid. Shake or mix well, and add 10 c.c. of ether. Pipette off 5 c.c. of ethereal extract into a white evaporating dish, and evaporate off ether. Add 1 c.c. of benzidene perborate solution, and another 1 c.c. if necessary. Positive reaction, intense blue.

STERCOBILIN.—A considerable amount of help in hepatic and intestinal disorders can very often be obtained by the test for stercobilin. The bilirubin and biliverdin of the bile gradually become reduced to stercobilin (intestinal urobilin) by the aid of bacteria. If a small portion is mixed in a test tube with three or four times the quantity of a saturated solution of perchloride of mercury a pink reaction, due to the formation of its mercuric compound, should result in about twenty minutes in normal stools. In diminished or total absence of bile this reaction is delayed or absent.

In the so-called saccharo-butyric form of intestinal fermentation the reaction is very marked. In diarrhœa, on the other hand, the pigments may be passed unchanged, and the reaction is green owing to the formation of mercuric hydro-bilirubin and biliverdin.

The value of the stercobilin fat and guaiac tests in certain instances of hepatic and pancreatic mischief, together with the microscopical findings, will be made clearer by the following table.

A great deal of work has been done in recent years on the bacterial flora of the intestine, and though much of it has so far proved fruitless, a considerable amount of knowledge of value has accrued from it.

Roughly speaking, about one-third of the solids of the stools consists of living and dead bacteria. The quantity is greater in diarrhœa and less in constipation. The number that can be cultivated is about 1 per cent., but this is not equivalent to the percentage of living bacteria, which is probably somewhat larger. The difficulty is that many of

| | Colour. | Re-action. | Odour. | Muscle Fibre. | Starch | Total Fats. % | Fats. Saponified. % | Fats. Unsaponified. % | Emulsion. | Saponification. | Stercobilin. | Urinary Urobilin. | Blood. |
|---|---|---|---|---|---|---|---|---|---|---|---|---|---|
| Obstruction of the bile duct. | White. | Acid, sometimes alk. | Putrid and sour. | O | O | 60 | 20 | 40 | - d | N | O | + | O[2] / + |
| Obstruction of pancreatic duct. | Pale Yellow. | Alk. sometimes acid. | Putrid. | + | + | 60 | 4–10 | 50–56 | N | O | N | O | O |
| Obstruction of both ducts. Ampulla of Vater | White. | — | Putrid. | + | + | 60 | 4–10 | 50–56 | O | O | O | + | O[3] / +[4] |
| Primary Pancreatic Achylia. | Pale. | — | — | O | O | 40 | 5 | 35 | N | O | N | O | O |
| Sprue[1] .. .. .. | Orange to Red. | Acid. | Faint acid. | + | + O | 34–57 | 29–32 | 5–28 | N | N | N | O | O |
| Normal .. .. .. | Dark Brown. | Amph. | Faecal | O | O | 25–30 | 12–15 | 12–15 | N | N | N | O | O |

[1] P. S. Cammidge. [2] Secondary cholangitis. [3] Stone—no wasting. Carcinoma—wasting and cachexia. [4] Malignant ulcer. Tissue wasting. Duodenal ulcer. N=normal; O=absent; +=excess.

The figures in the above table are approximate only.

In primary pancreatic achylia there may be the absence of one or more of the proteolytic or amylolytic ferments and of lipase. The absence of the latter alone is the type given in the figure.

the anaërobic organisms do not survive attempts at cultivation.

Though the number of varieties is large, certain types are found to predominate. By making a thin transparent film as detailed above, and staining this with gram and counter-staining with a weak acid fuchsin solution, a very fair estimate of their nature and proportions can be made. In order to obtain uniform results the gram stain should be applied for four minutes, the iodine for half a minute, and the alcohol for one minute : if this is not done confusion may result.

In an ordinary healthy adult on a mixed diet the ratio of gram +ve and gram —ve organisms is about equal. The preponderating gram +ve bacteria are the *B. bifidus* and *B. acidiphilus,* which both attack carbohydrate food or their cleavage products, and the large and small subtiloid bacilli, which attack proteids and their products. The chief gram —ve organisms are the *B. coli* group with the *B. lactis aërogenes,* the *B. coli* having the largest representation of any form.

Besides these there are many others, bacilli and cocci and spores. The *streptococcus fæcalis,* described by Andrews, is almost always present.

Most of the bacteria referred to above are in the proportions present perfectly harmless. There is an acquired and an hereditary immunity, and the liver has probably large detoxicating powers. They are even beneficial, for they inhibit the growth of pathogenic forms. Under certain circumstances, however, as in stasis following obstruction, the *B. coli,* for instance, may get out of hand, and along with the others become extremely toxic through their products.

It is not, however, only in acute disease that they may prove harmful to these parts, for carbohydrate and protein cleavage may through their influence or through that of extraneous organisms become a cause of chronic mischief.

Herter, whose work in this respect is second to none, differentiates three main types of ultra or vicious cleavage, and his method of classification has now been adopted by all workers on the subject.

THE INDOLIC TYPE.—This is characterised by excessive proteolytic decomposition caused by members of the *B. coli* group and probably *B. putrificus*. In the gram-stained field the gram —ve bacteria predominate. The urine gives a strong indican reaction.

The ratio of $\frac{\textit{ethereal sulphates}}{\textit{inorganic sulphates}}$ in the urine, instead of the normal $\frac{1}{10}$ becomes $\frac{1}{5}$ or $\frac{1}{4}$, and occasionally the indol acetic reaction is strongly +ve (red colourisation of urine on addition of HCl, and if necessary a few drops of 2 per cent. sodium nitrite).

THE SACCHARO-BUTYRIC form of fermentation is due to the presence and action of a very large number of anaërobic butyric acid producing bacteria in the lower ileum and the colon. The chief organism concerned is the *B. aërogenes capsulatus* (gram +ve). Hence a gram field in which the gram +ve predominate. The stercobilin reaction is very marked.

THE COMBINED INDOLIC AND SACCHARO BUTYRIC TYPE.—This form leads to rapid fall in health accompanied by neurasthenia. Herter and Kendal have shown how it is possible to modify the flora by alteration in the diet. On a protein diet the coli group (gram —ve) were shown to be markedly predominant, and both large and small subtiloid gram +ve bacteria were present in fair numbers. The ratio gram +ve and gram —ve was about even.

But on a carbohydrate diet gram +ve organisms became markedly predominant, the *B. coli* (—ve) diminishing very greatly and the *B. acidiphilus* (—ve) and *B. bifidus* (+ve)[1] becoming prominent. The significant fact was that this occurred irrespective of the normal feeding habits of the animal, for it was manifested equally in carnivorous and herbivorous animals. Treatment based on these researches offers a very fair field for advance, and some promising results have already been published of the treatment of cases of rheumatoid arthritis, colitis and pernicious anæmia by starving the offending organism. But much more work on the subject is required, and it is to be hoped that by adopting these lines our knowledge and treatment of many chronic diseases may be furthered

### REFERENCES.

GENERAL.

Schmidt and Strasburger : *Die Fæces des Menschen.* 1905.

Sahli's *Diagnostic Methods.* 1911.

Harley and Goodbody : *Chemical Investigation of Gastric and Intestinal Diseases.* 1906.

O. T. Williams : *Liverpool Med.-Chir. J.*, 1911, xxxi. 362.

*Proc. Roy. Soc. Med.* : Discussion on " Alimentary Toxæmia," vol. vi. 1913.

OCCULT BLOOD.

A. J. Clark : *St. Bartholomew's Hospital Reports*, vol. 45, 1909, p. 97.

Colwell and MacCormac : *Arch. of Middlesex Hospital*, vol. 23, 1911.

FATS.

Begg : *Sprue, its Diagnosis and Treatment*, 1912.

Garrod and Hurtley : *Quart. J. Med.*, 1912–13, vi. 242.

BACTERIOLOGY.

Herter : *Common Bacterial Infections of the Digestive Tract*, 1907.

Herter : *Lectures on Chemical Pathology*, 1902.

Herter and Kendal : *J. Biol. Chem.*, 1909, x. 203.

Andrews and Horder : *Lancet*, 1906, vol. ii. p. 708.

[1] A slight indol acetic reaction was found to be rarely absent in seven or eight hundred investigations of the urine of patients suffering from chronic disease at the Royal Mineral Water Hospital, but it is not often very marked.

It is sometimes intense in acute disease, appearing and disappearing very suddenly. In a case of pneumonia it suddenly appeared on the day after the crisis, and disappeared completely in five days.

